# Quantitative Study of Porosity and Pore Features in Moldavites by Means of X-ray Micro-CT

**DOI:** 10.3390/ma7043319

**Published:** 2014-04-24

**Authors:** Giovanni Pratesi, Stefano Caporali, Francesca Loglio, Gabriele Giuli, Lenka Dziková, Roman Skála

**Affiliations:** 1 Museo di Storia Naturale, Università di Firenze, Via G. La Pira 4, 50121 Firenze, Italy; E-Mail: g.pratesi@unifi.it; 2 Department of Chemistry, Università di Firenze, Via della Lastruccia 3, 50019 Sesto Fiorentino, Italy; 3 Centro di Cristallografia Strutturale, Università di Firenze, Via della Lastruccia 3, 50019 Sesto Fiorentino, Italy; E-Mail: francesca.loglio@unifi.it; 4 Scuola di Scienze e Tecnologie, Sezione di Geologia, Università di Camerino, Via Gentile III da Varano, 62032 Camerino, Italy; E-Mail: gabriele.giuli@unicam.it; 5 Department of Geological Sciences, Masaryk University, Kotlářská 2, CZ-61137 Brno, Czech Republic; E-Mail: dzikova@sci.muni.cz; 6 Institute of Geology, Academy of Sciences of the Czech Republic, v.v.i., Rozvojová 269, CZ-16500 Praha 6, Czech Republic; E-Mail: skala@gli.cas.cz

**Keywords:** tomography, tektites, moldavites, Muong Nong, plastic deformation

## Abstract

X-ray micro-computer aided tomography (μ-CT), together with optical microscopy and imaging, have been applied to the study of six moldavite samples. These techniques enabled a complete characterization to be made of the textural features of both Muong Nong-type and common splashform moldavites. A detailed study of the size and distribution of pores or bubbles confirmed the marked variability in pore size among the samples, as well as within each sample, and indicated in the Muong Nong-type moldavites the presence of at least two deformation stages which occurred before and after pore formation.

## 1. Introduction

Tektites are natural acid silicate glasses that occur in geographically limited areas, so-called strewn fields. They differ from common volcanic glasses in both chemical and physical properties [1]. Their characteristic features include high silica and very low water content, the presence of lechatelierite (amorphous SiO_2_) particles, and a low Fe^3+^/(Fe^2+^ + Fe^3+^) ratio [2–8]. Tektites are today generally considered to be products of hypervelocity impacts on Earth; usually they experienced aerodynamic transport and can be deposited at great distances (up to several hundred kilometers) from the presumed source crater. Generally, several morphologically different types of tektites may be recognized: (i) most common are the splashform tektites, found in each of the known strewn fields; (ii) aerodynamically shaped tektites are button-like objects occurring exclusively in Australia; (iii) micro-tektites; and (iv) Muong Nong-type tektites (the rarest type). The Muong Nong-type tektites are named after the city of Muong Nong in Laos where they were first described by Lacroix [9]. Typical features of the archetype Muong Nong-type tektites include blocky shape, highly variable size and weight, layered structure, high content of crystalline inclusions [10–12] and high porosity. Chemical characteristics that distinguish the Muong Nong-type tektites from splashform counterparts include an overall chemical heterogeneity, high volatile trace element abundances [13] and high water content [14,15].

Moldavites (aka Central European Tektites; CET) are thought to represent distal ejecta formed during the impact event that produced the 24-km diameter Ries crater in Germany. They have been found at distances of 200 km to 450 km from the point of impact in the Czech Republic [16], Austria [17], and Germany [18]. The chemical, physical, and morphological properties of moldavites, as well as the data on their geographical distribution, have been reported in numerous papers and books, e.g., [19–29]. Although the link between the Ries crater and moldavites has been generally accepted [26,30–32], the exact formation process and source materials remain a matter of debate [13,27,33–40]. The age of moldavites, as the age of the Ries crater, has been the subject of numerous papers [41–43]. Most recently, Di Vincenzo and Skála [44] and Buchner *et al.* [45] concluded that the age of moldavites and the Ries structure is 14.68 ± 0.11 Ma and 14.59 ± 0.20 Ma, respectively.

Although the vast majority of moldavites are of the splashform type, some authors [46–49] have described Muong Nong–type moldavites from the South Bohemian partial strewn field. The Muong Nong-type moldavites, contrary to the generally larger and considerably blocky Muong Nong-type tektites from Asia, are of much smaller size, similar to splashform moldavites. In some rare cases, some Muong Nong-type moldavites consist of two types of glass, porous and compact, resulting in a layered appearance, which is considered the typical feature of Asian Muong Nong-type tektites. In most cases, however, only the porous layer of a Muong Nong-type moldavite is preserved separately. This highly specific type of moldavite also displays high structural heterogeneity with high content of lechatelierite inclusions, high pore density, and substantial chemical heterogeneity. Since the Muong Nong-type tektites, in general, may carry important information on the tektite-forming process [13], we decided to characterize the internal structure of four Muong Nong-type moldavites and compare the pore features and the porosity degree with that of two splashform moldavites. We used X-ray micro-computer aided tomography (μ-CT) to extract the 3D pore features on representative portions (about 400–500 mm^3^) of the samples. This is a relatively new technique allowing the accurate evaluation of the size, shape and distribution of different features of a sample characterized by different X-ray absorption coefficients; in this particular case the micro- and macro-pores or bubbles of the tektites. Since Ketcham and Carlson [50] first proposed the use of μ-CT to extract 3D features of geological materials, many researchers in the field have applied this technique using either synchrotron or laboratory X-ray sources [51,52]. The technique has also been shown to be a useful tool to study meteorites [53], especially chondrites. The texture and distribution of mineral phases [54–57], the shape of chondrules [58], the distribution of pores [59–61], and the different mineralogical phase distribution of chondrites were investigated in order to assess the technique as a possible non-destructive tool for the classification of ordinary chondrites [62]. The technique of μ-CT has also been successfully applied to the study of other planetary related materials, such as the Stardust impact tracks [63,64], the silicate inclusions in meteoritic chromite grains [65] and splashform moldavites [66].

## 2. Materials and Methods

### 2.1. Samples

**Figure 1.**
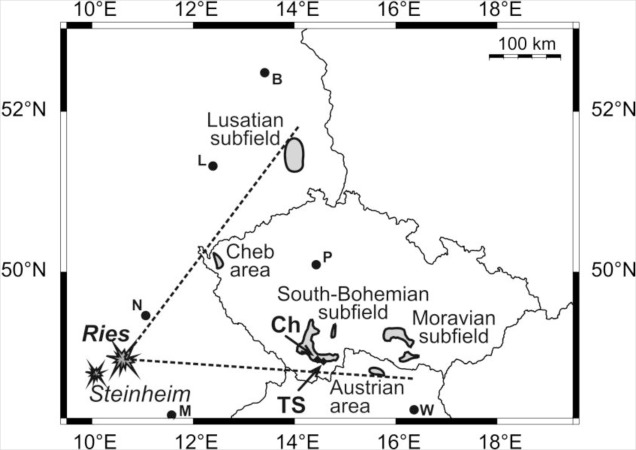
Map showing the extent of known moldavite occurrences (grey shading) and the location of the studied moldavites: Ch = Chlum; TS = Trhové Sviny. Also shown are the impact craters Ries and Steinheim. (P = Prague, M = Munich, N = Nuremberg, L = Leipzig, B = Berlin and W = Vienna).

A total of six moldavite samples from the South Bohemian substrewn field (Czech Republic) were selected to be investigated by X-ray micro-computer aided tomography and polarized light microscopy. In particular, the samples Chlum 1 and Chlum 2 (splashform moldavites, chips of about 6 mm × 5 mm × 3 mm) originate from a locality near the village of Chlum nad Malší, 7 km NNE of Kaplice (48°48′2.049″ N, 14°30′52.602″ E), and the four Muong Nong-type moldavite samples (Nos. 2, 7, 8, 23, slices of about 10 mm × 10 mm × 0.8 mm) originate from the village of Slávče, 5 km WSW of Trhové Sviny (48°48′10.887″ N, 14°37′5.851″ E; Figure 1).

### 2.2. X-ray Micro Computer Aided Tomography (μ-CT) and 3D Data Extraction

Micro-CT data were collected using a Skyscan 1172 high-resolution MicroCT system at the University of Florence (CRIST). This system has a sealed, microfocus tungsten X-ray tube with a 5 μm focal spot size operating at 100 kV, 100 μA, and a 11 Mpixel detector panel with a 16 bit pixel depth. The sample was placed on the pedestal between the X-ray source and the CCD detector, recording the 2D X-ray images by rotating the sample 180° with a slice-to-slice rotation angle of 0.2°. In this way, nine hundred 2D images were recorded for each sample. The total acquisition time was approximately 40 min. Spatial resolution of the single tomogram was kept in a range of 5–10 μm in terms of pixel size, giving a corresponding voxel resolution range between 125 and 1000 μm^3^.

The 3D image of the object’s internal structure was reconstructed using a modified Feldkamp algorithm for cone-beam acquisition geometry realized in Nrecon v.1.6.3.3 software (Bruker-MicroCT, Kontich, Belgium). The final images were reduced to 8 bit pixel depth. The alignment, beam-hardening and ring artifact corrections were made before starting the reconstruction process.

**Figure 2.**
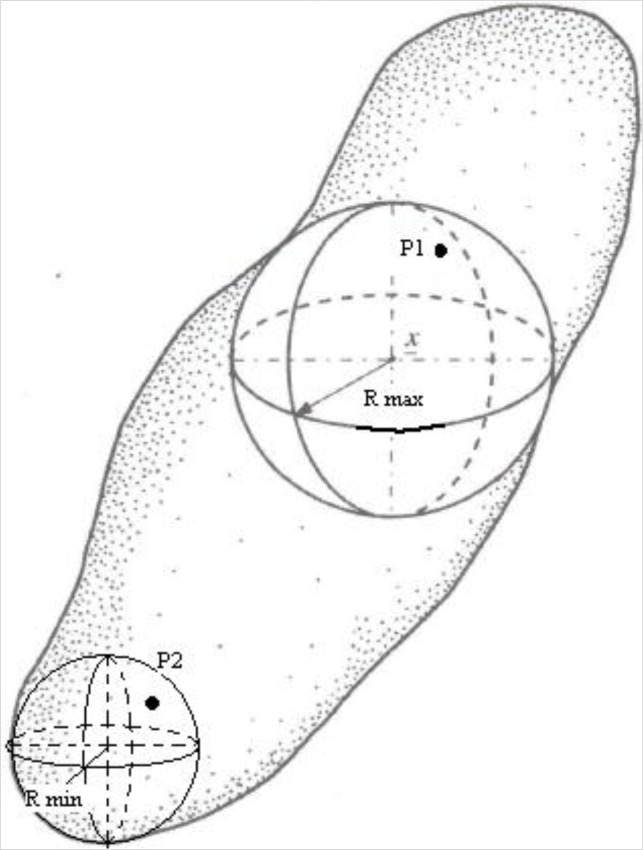
Example of local thickness for two points (P_1_ and P_2_) within an irregularly shaped pore determined by fitting maximal spheres. All the points within a spherical pore are characterized by the same radius. *Vice versa*, irregularly shaped pores such as that depicted, are characterized by different radii. Modified from [67].

Morphometric parameters were calculated in 3D on a surface-rendered volume model by using CT-analyzer software v.1.11 (Bruker-MicroCT, Kontich, Belgium). In particular, all calculations were performed on segmented images over selected volumes of interest (VOI) within the tektites. The total volume, density and surface of the pores inside the tektites were calculated on the marching cubes volume model of the pores binarized as solid objects, inside the selected volumes of interest. Average pore diameter was evaluated using the definition of the local thickness for a point within a solid given by Hildebrand and Rüegsegger [67], which is the diameter of the largest sphere that fulfills two conditions: the sphere encloses the point (but the point is not necessarily the center of the sphere) and the sphere is entirely bounded within the solid surfaces (Figure 2).

In this way the calculated average pore diameter is independent of the object shape. The method that the CT-analyzer uses starts with a “skeletonization” identifying the medial axes of all structures. Then the “sphere-fitting” local thickness measurement is made for all the voxels lying along this axis. The average pore size was larger than the effective pixel size in micro-CT reconstruction and larger than the spot size of the X-ray source.

The visualization of the 3D CT images was created using a model built with a marching cubes 33 algorithm; surface-rendered 3D model of the whole object and the pores were loaded with CT-Vol v.2.1 software (Bruker-MicroCT, Kontich, Belgium). The images of the whole tektites were colored and shown as gray solids or, alternatively, colored images of solid pores were overlaid with the tektites VOI to visualize the pore distribution inside the tektites. All the images were smoothed with the Skyscan software (Bruker-MicroCT, Kontich, Belgium).

### 2.3. Polarized Light Microscopy

To complement the characterization of the moldavite texture obtained through μ-CT analysis, particularly to retrieve information on strain that cannot be revealed by X-ray tomography, optical microscopy and imaging were performed at the laboratories of the Museo di Scienze Planetarie (Prato) by means of an Axioplan-2 polarizing optical microscope equipped with Axiocam-HR camera and Axiovision 4.1 software (Carl Zeiss Microscopy GmbH, Jena, Germany).

## 3. Results

**Figure 3.**
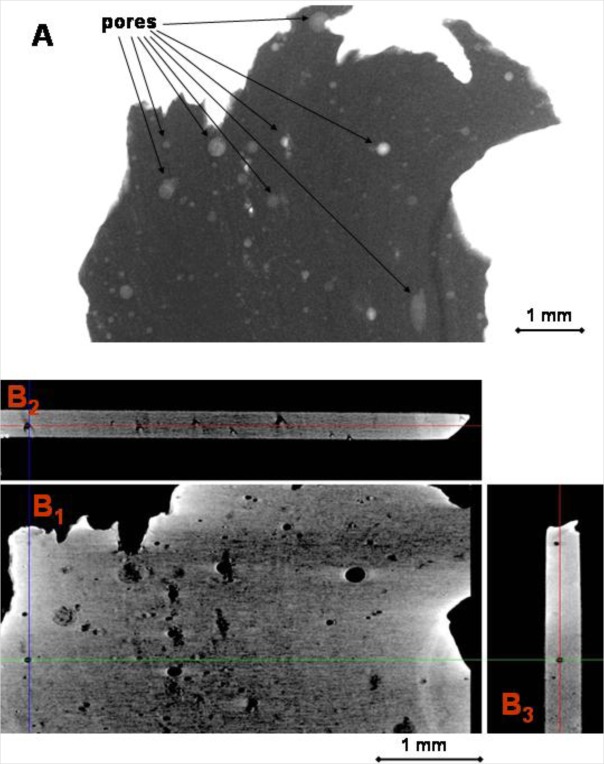
(**A**) a typical X-ray transmission image of a tektite (sample No. 2), located in the microCT sample holder. Pores, some of which are indicated by arrows, are detectable as lighter gray spots; (**B**) reconstructed slices of the VOI (volume of interest) along the (**B_1_**) transaxial, (**B_2_**) coronal and (**B_3_**) sagittal planes.

Figure 3A shows a typical X-ray transmission image of a tektite (sample No. 2), located in the microCT sample holder. In this case, pixels of material that have a large X-ray attenuation are dark, whereas pores characterized by lower X-ray attenuation are detectable as light gray spots.

A pre-analysis was made after reconstructing a dataset of each sample, loading it into DataViewer and checking for abnormalities or irregularities that may have occurred during image reconstruction. Three-dimensional portions of materials (VOI, volume of interest) were chosen with the objective of selecting as much volume as possible, avoiding the portions affected by unavoidable irregularities, *i.e.*, beam hardening effect or other artifacts.

The VOI was selected to enable a statistical analysis of the pore size distribution and number to be made. This procedure also allows the largest possible portion of the sample to be selected, but avoids the edges where beam hardening or ring artifacts could lead to misevaluation of the pores. In order to compare the data of samples of different sizes, all the morphometric parameters were normalized with respect to the relative VOI. Tomographic slices, obtained after data reduction and artifact removal on a selected portion (VOI) of the same sample, and are also displayed in Figure 3B. In this case, pixels of material showing a large X-ray attenuation are light gray, whereas pores characterized by lower X-ray attenuation are detectable as dark spots. At each 3D location (shown at the cross-hairs), three orthogonal views are retrieved from the whole dataset as shown:

- transaxial view (the normal images, in *x*-*y* plane), (B_1_);
- coronal view (*x*-*z* plane), (B_2_);
- sagittal view (*z*-*y* plane). (B_3_) Sub-millimeter sized pores, characterized by sharp edges, are clearly detectable in all the images.

**Figure 4.**
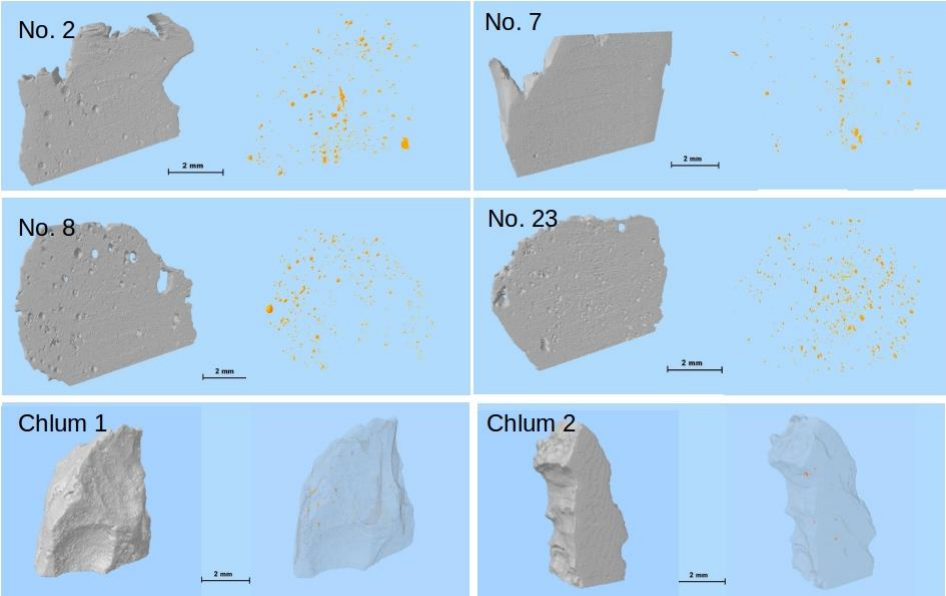
Three-dimensional rendered images of the analyzed moldavites. The sample surfaces are displayed in the left images while, on the right, the isosurface images highlight the closed pores present inside the samples.

Figure 4 displays the isosurface 3D reconstructions of the tektite surface (left) and their pores (right). In particular, the figure displays the three-dimensional distribution of pores (in orange) across the volume of interest (VOI). It is clear that the Muong Nong-type moldavites studied in this work display larger and more numerous pores compared to the splashform samples (Chlum 1 and Chlum 2). Although pores of larger size have been observed in other spashform-like moldavites [66], the total porosity of these samples was always lower than those of Muong Nong, suggesting that higher porosity levels must be considered a peculiar feature of the Muong Nong-like.

**Figure 5.**
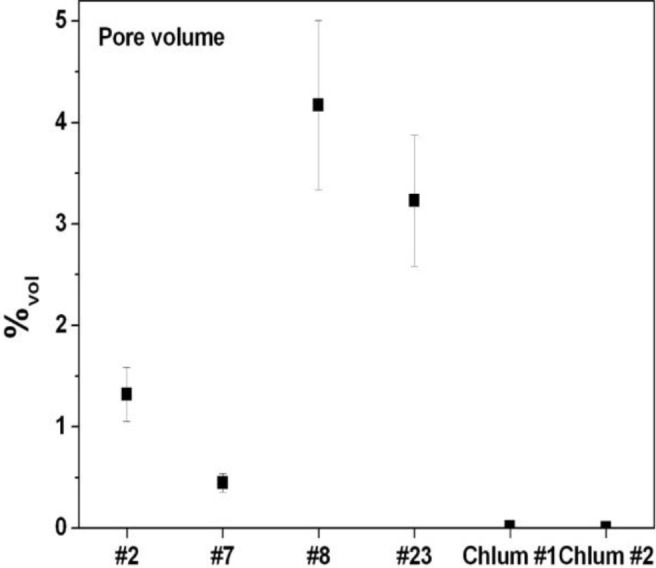
The porosity of the samples is expressed as pore volume percentage. It should be noted that splashform moldavites are characterized by very low porosity (Chlum 1 and Chlum 2 displaying 0.011% and 0.006%, respectively), whereas the Muong Nong-types have much higher values.

**Figure 6.**
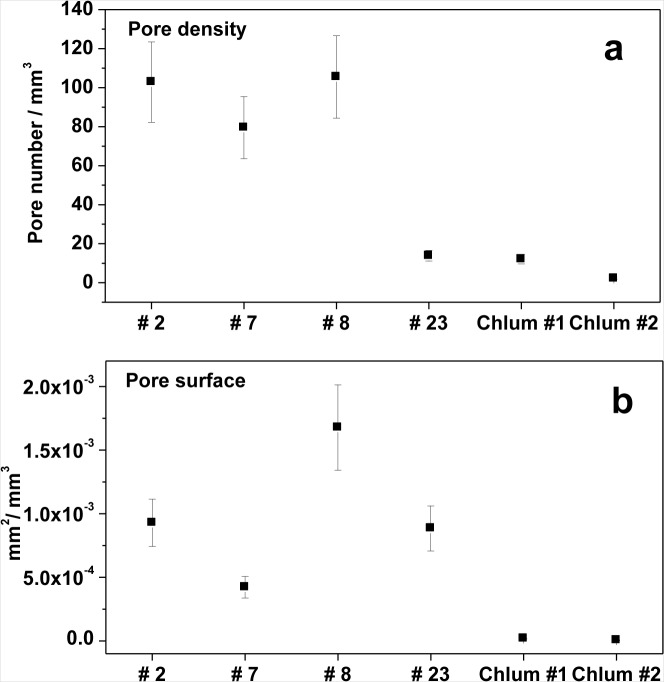
(**a**) Pore density (number of pores per mm^3^) and (**b**) pore surface density of the investigated samples. Error bars ±20%.

**Figure 7.**
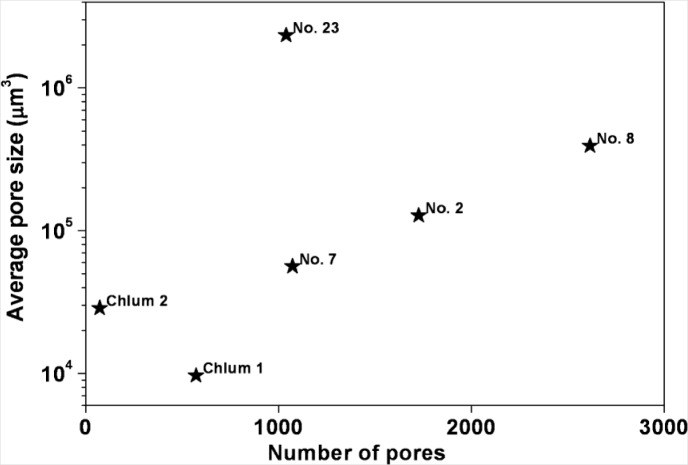
Semi-logarithmic plot of the average pore size *versus* the number of pores. It is evident that splashform moldavites (Chlum 1 and Chlum 2) are characterized by relatively few small pores compared to the Muong Nong-type.

Figure 5 plots the porosity (volume% of pores) determined for the six studied samples. Among the Muong Nong-type moldavites, sample 7 shows the lowest degree of porosity, well below 1%, the other samples are characterized by much larger values up to more than 4% (sample No. 8). In agreement with previous reports [66], splashform moldavites were found to have very low porosity, as exemplified by Chlum 1 (0.011%) and Chlum 2 (0.006%). Substantial differences were observed in the average number of pores per mm^3^ (Figure 6). Muong Nong-type moldavite sample No. 7, even though characterized by a low overall porosity, has a very high number of pores, about 100/mm^3^, similar to the more porous samples No. 2 and 8. Clearly, sample No. 7 contains a large number of very tiny pores, which is highlighted below. Unusual features were also detected for the samples No. 23 and Chlum 1; despite their very different degree of porosity, both display only a relatively low number of pores (about 10/mm^3^). That is due to the pronounced difference in pore size; tiny pores in Chlum 1 (splashform-type) and much larger pores in No. 23 (Muong Nong-type). The pore size differences are also highlighted in Figure 7, where the average pore size is plotted *versus* the number of detected pores. Sample No. 23 and Chlum 1 display the largest and smallest average pore size, respectively.

Another useful parameter that provides quantitative information on the type of pores present is the pore surface. It has been calculated as the total surface of closed pores with respect to the VOI volume (Figure 6B). Since only pores that are not intersected by the VOI surfaces (closed pores) were taken into account for the evaluation of pore surface, the values obtained are underestimated for all the samples. Nevertheless, a statistical qualitative indication of the pore shape can be achieved by evaluating the calculated pore surface as a function of the number of closed pores. Small or irregular pores result in a larger calculated pore surface, whereas spherical or subspherical pores translate into smaller values of pore surface. It is interesting to note that sample No. 8 shows the highest value, whereas sample No. 2 is characterized by a much lower surface, about 9 × 10^−3^ mm^2^/mm^3^. Considering that the number of pores is nearly the same for these two samples, it is evident that the pores present in sample No. 8 are larger and less spherical than those present in sample No. 2.

**Figure 8.**
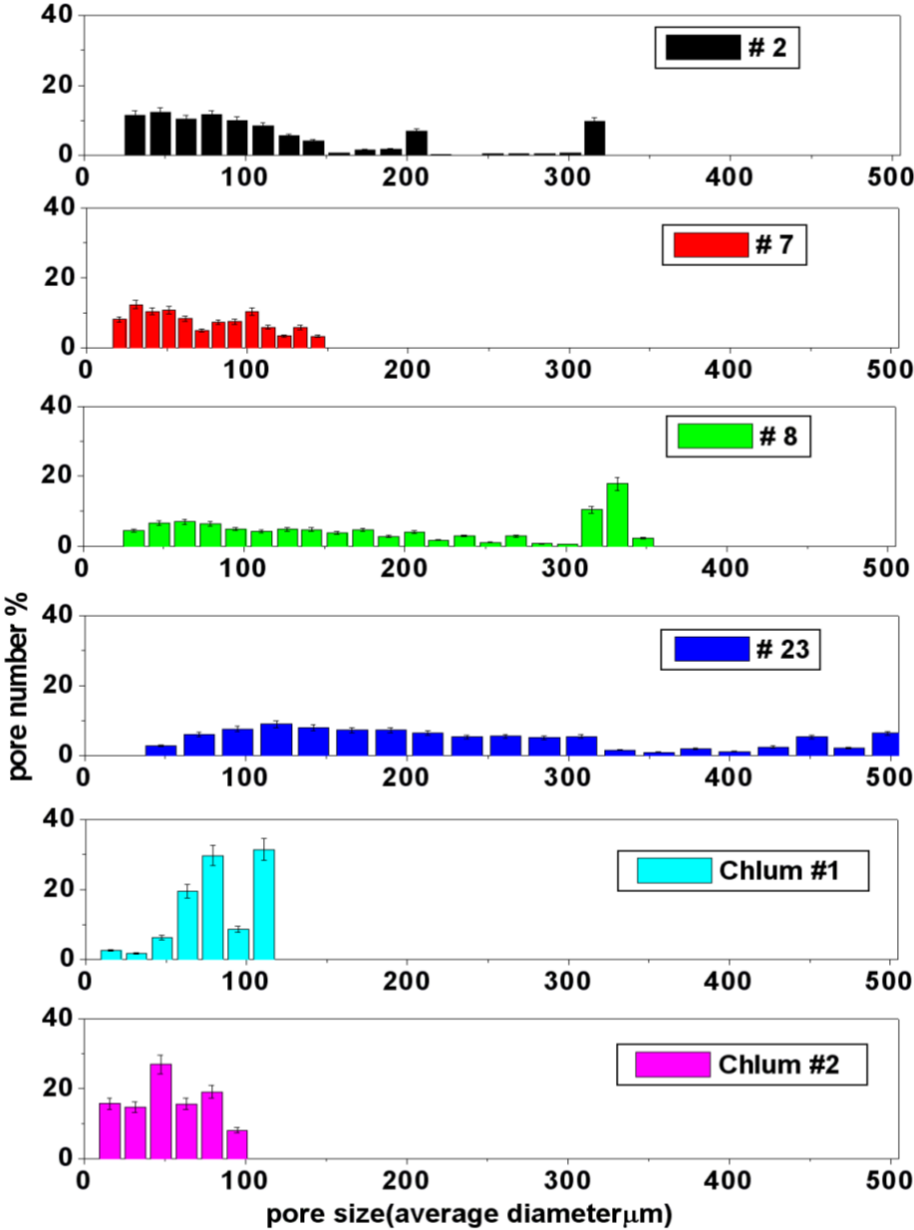
Pore size distribution for the six samples analyzed. Error bars ± 20%.

The pore size distribution is shown in Figure 8. The histograms clearly illustrate that there is a large range of pore size distribution among the investigated samples. The largest pores in the splashform moldavites Chlum 1 and Chlum 2 do not exceed 100 μm; the largest pores in the Muong Nong-type moldavite No. 7 do not exceed 150 μm and the largest pores overall are found in sample No. 23 reaching 500 μm. Sample No. 8 is characterized by a large number of large pores, *i.e.*, almost 20% of the pores show diameters between 300 and 350 μm. Table 1 summarizes the data extracted from the porosity analysis.

The studied moldavites also differ on a macroscopic scale. The two splashform moldavites are transparent or translucent, green colored, and rather compact; whereas those of the Muong Nong-type are translucent on the edges only, display generally much darker colors, seem to be composed of various domains (sometimes layer-like), and contain an appreciable amount of pores and pits that possibly represent open pores.

**Figure 9.**
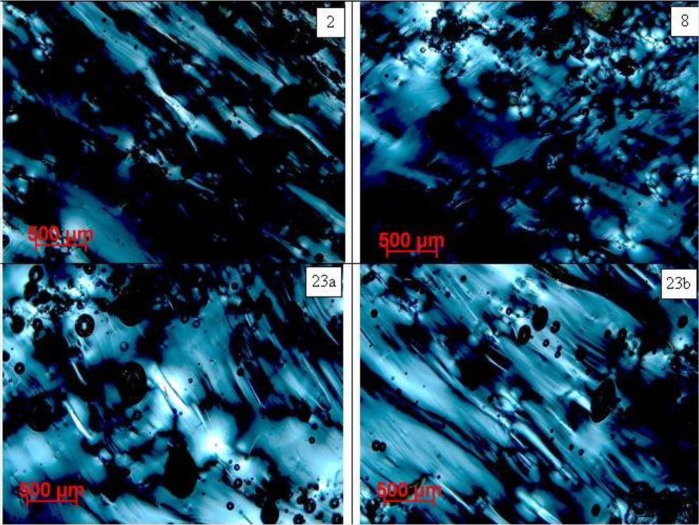
Optical images (crossed polarized lenses) showing the difference in the strain birefringence as well as the pore shape exhibited by samples No. 2, No. 8 and No. 23. Residual strain is revealed by the flow lines mainly oriented NW-SE, while spherical or elongated pores are detectable throughout the images.

**Figure 10.**
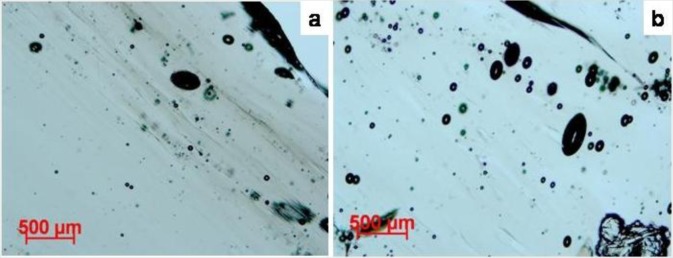
Optical images showing the elongated shape of the pores in sample (**a**) No. 7 and (**b**) No. 23. The pore elongation clearly differs from the flow lines (oriented NW-SE).

In thin sections, both types are more or less transparent, lightly yellow, yellow-green or yellow-brown colored. The main difference is the presence of at least two intercalated or interwoven melts differing in color and abundance of lechatelierite inclusions and frequency of pores in Muong Nong-type moldavites (see Figures 9 and 10). On the contrary, the splashform moldavites tend to be rather homogeneous. Glass domains in Muong Nong-type moldavites usually form schlieren of various shapes and widths. Furthermore, their pores have very variable sizes and shapes. It is also quite common that individual variably-sized pores aggregate into larger clusters. Between crossed polarizers moldavite glass displays anomalous behavior showing local strong birefringence (Figure 9). Birefringent domains usually occur close to or along the schlieren or lechatelierite inclusions and pores; they are much more frequent in Muong Nong-type moldavites than splashforms.

## 4. Discussion

The application of X-ray micro-computer aided tomography has allowed a statistical analysis to be conducted with high spatial and contrast resolution of a large number of pores of different sizes present inside six moldavite samples on large and representative portions of the samples (about 400–500 mm^2^). The moldavite samples investigated in this study show highly variable pore content and distribution (Figures 4 and 5), as well as considerable differences in pore shape (Figure 6). The pore sizes display a general trend: the amount of porosity correlates to larger pore sizes (e.g., samples No. 7, Chlum 1 and Chlum 2 show few, small pores). Conversely, in samples No. 8 and No. 23, that have the highest porosity, the pores reach the largest sizes (Figures 4, 5, 7 and 8). This trend is evident from the diagram in Figure 7.

Matsuda *et al.* [68] argued that pores formed as a result of gas expansion inside the glass, without being followed by deformation processes, should be spherical. The tomography images (Figure 3) as well as optical images (Figures 9 and 10) show that in the moldavite samples studied many pores, in particular the largest ones, are not spherical. This suggests that a further deformation of moldavites occurred after pore formation.

It is also important to note the mutual relationship among the pores of the schlieren and residual strain. According to Engelhardt and coworkers [27], each individual moldavite body represents a population of chemically different primary units. The schlieren texture, in the moldavite samples, may represent two subsequent rheological regimes that can be distinguished during the cooling of moldavite bodies, when the temperature is too low to allow mixing. Fluid particles of different composition and viscosity may be extended into thin lamellae in a stress field of laminar flow. At lower temperatures, these arrays were subsequently plastically deformed (also under conditions of compressional stress), probably concomitant with the shaping of individual moldavite bodies [27].

Neither schlieren nor strain can be revealed by X-ray tomography. Consequently, polarized light microscopy was used to observe these features. As reported by Majmundar and O’Keefe [69], the tektites display an overall strain pattern as well as that associated with the notches. The strain pattern corresponds to that expected for bodies which were rapidly cooled and, therefore, shows differences between the outer and the inner portions of the bodies: compressional strain in a tangential direction and tensional in the radial direction. Moreover the anomalous strain-induced birefringence may enhance textures connected with the laminar flow as well as those related to plastic deformation. The strain-induced birefringence images of the moldavites are shown in Figure 9. They demonstrate a marked difference among the individual investigated samples, although the strain generally seems to be directly proportional to the porosity. Another interesting feature revealed by Figure 9 is the prevalence of laminar and chaotic flow. The laminar texture is most developed in sample No. 7, whereas it is less apparent in the other samples. On the contrary, significant chaotic flow fabric is evident in sample No. 8. In general, the Muong Nong-type moldavites display more pronounced birefringence, possibly illustrating the presence of much more heterogeneous strain patterns due to considerably higher heterogeneity compared to the splashform moldavites.

The distribution of pores is generally irregular. In most cases, the pores lack any obvious preferential alignment with respect to flow textures. Only locally, the pores seem to be concentrated along the schlieren.

Most of the pores are spherically shaped, however, in sample No. 7 and No. 23 certain pores of different sizes, including the smallest ones, are elongated (Figure 10). It should be noted that elongation axes of the pores in these samples differ in their inclination with respect to the direction of the laminar schlieren. Specifically, in sample No. 7 the pore elongation axes are inclined by about 30 degrees from the direction of the schlieren, whereas in sample No. 23 there are at least two groups of pores whose elongation axes show departures of about 35 and 70 degrees from the direction of the schlieren. Large inclination of the elongation axes of the latter group of pores from the direction of the flow texture indicates that they formed in a completely different stress field compared to the schlieren pattern. Since the pores distinctly displace the thin layers of the schlieren themselves, it is clear that their formation post-dated the formation of flow textures.

On the basis of our observations a possible scenario for the formation of the pores in Muong Nong-type moldavites can be made.

The moldavites history is certainly related to the Ries crater. Regardless of the exact formation process (impact fusion of terrestrial sedimentary material, vapor fractionation of a relatively homogeneous melt, radiation melting of surface sediments, volatilization of elements and formation via plasma), each individual moldavite body was characterized by chemically different primary units destined to experience dissimilar regimes of stress. As shown in this work, at least two regimes are demonstrated by the relationship between schlieren and pores. Firstly, the schlieren was generated by the occurrence of inhomogeneity (probably fluids with different compositions and viscosity [70]) extended into lamellae in a stress field of laminar flow. Subsequently, as the tektites moved upwards, the temperature and the pressure decreased allowing the growth of the pores that, sometimes, displaced schlieren and were plastically deformed by the same force that shaped the individual moldavites body.

The appearance and relative abundance of the pores represent useful features to distinguish between splashform and Muong Nong-type moldavites. Pores in Muong Nong-type moldavites are much more numerous than in splashforms. Furthermore, in Muong Nong-type moldavites observed the pores agglomerate into clusters, which in limiting cases may result in the so-called frothy appearance absent in their splashform counterparts. The higher values of porosity in Muong Nong-type clearly indicate that the degassing process had not reached completion during the cooling of these tektites.

## 5. Conclusions

Combined X-ray micro computer aided tomography (μ-CT) and polarizing optical microscopic study of two splashform and four Muong Nong-type moldavites allowed the detailed characterization of the pore and strain distribution in this type of material. The study revealed the marked variability in pore size, distribution and shape between and within the individual samples. Moreover, the density and distribution of the pores confirmed that it is possible to distinguish between normal and Muong Nong-type moldavites using these parameters. Comparison of the directions of flow texture with those of the elongation axes of certain pores in Muong Nong-type moldavites revealed that the formation of at least a part of the pores post-dates the origin of the schlieren pattern.

## Figures and Tables

**Table 1.** Porosity analysis results of six tektites.

Sample	Unit	No. 2	No. 7	No. 8	No. 23	Chlum 1	Chlum 2
Total volume (VOI)	μm^3^	1.68 × 10^10^	1.35 × 10^10^	2.48 × 10^10^	7.53 × 10^10^	4.76 × 10^10^	3.36 × 10^10^
Porosity(pore volume)	%	1.32	0.45	4.17	3.23	0.011	0.006
Average pore number	Number of pores/mm^3^	102.9	79.5	105.5	13.8	12	2.1
Average pore surface density	mm^2^/mm^3^	9.2 × 10^−4^	4.2 × 10^−4^	16 × 10^−4^	8.8 × 10^−4^	0.18 × 10^−4^	0.05 × 10^−4^
Average pore size	μm^3^	0.13 × 10^6^	0.5 × 10^6^	0.4 × 10^6^	2.34 × 10^6^	0.009 × 10^6^	0.03 × 10^6^
